# No association between SCN9A and monogenic human epilepsy disorders

**DOI:** 10.1371/journal.pgen.1009161

**Published:** 2020-11-20

**Authors:** James Fasham, Joseph S. Leslie, Jamie W. Harrison, James Deline, Katie B. Williams, Ashley Kuhl, Jessica Scott Schwoerer, Harold E. Cross, Andrew H. Crosby, Emma L. Baple

**Affiliations:** 1 RILD Wellcome Wolfson Centre, University of Exeter Medical School, Royal Devon & Exeter NHS Foundation Trust, Barrack Road, Exeter, United Kingdom; 2 Peninsula Clinical Genetics Service, Royal Devon & Exeter Hospital, Gladstone Road, Exeter, United Kingdom; 3 University of Exeter, Department of Biosciences, Exeter, United Kingdom; 4 Center for Special Children, La Farge Medical Clinic-VMH, La Farge, Wisconsin, United States of America; 5 Department of Pediatrics, University of Wisconsin, Madison, Wisconsin, United States of America; 6 Department of Ophthalmology, University of Arizona College of Medicine, Tucson, Arizona, United States of America; HudsonAlpha Institute for Biotechnology, UNITED STATES

## Abstract

Many studies have demonstrated the clinical utility and importance of epilepsy gene panel testing to confirm the specific aetiology of disease, enable appropriate therapeutic interventions, and inform accurate family counselling. Previously, *SCN9A* gene variants, in particular a c.1921A>T p.(Asn641Tyr) substitution, have been identified as a likely autosomal dominant cause of febrile seizures/febrile seizures plus and other monogenic seizure phenotypes indistinguishable from those associated with *SCN1A*, leading to inclusion of *SCN9A* on epilepsy gene testing panels. Here we present serendipitous findings of genetic studies that identify the *SCN9A* c.1921A>T p.(Asn641Tyr) variant at high frequency in the Amish community in the absence of such seizure phenotypes. Together with findings in UK Biobank these data refute an association of *SCN9A* with epilepsy, which has important clinical diagnostic implications.

## Author summary

Epilepsy is defined as a tendency to have seizures, affecting around 1:100 people worldwide. Some genetic types of epilepsy can be diagnosed using a test that examines genes that have previously been shown to cause epilepsy when affected by genetic alterations. Identifying the genetic cause of a patient’s epilepsy can help determine which treatments are likely to be helpful, provide prognostic information and inform accurate family counselling. The genes that are included in diagnostic epilepsy gene testing panels are decided by reviewing published scientific studies. Previous publications have described an association between the *SCN9A* gene and forms of epilepsy, leading to inclusion of the *SCN9A* gene in diagnostic testing panels. In this paper we present the results of our genetic findings and follow-up studies of *SCN9A* gene alterations in the Amish community and UK Biobank, alongside a fresh appraisal of previous studies investigating the role of *SCN9A* in epilepsy. Together our findings strongly refute an association between SCN9A and epilepsy. This work has important clinical implications and should lead to the re-evaluation of *SCN9A* by expert groups and its removal from genetic testing panels, preventing future genetic misdiagnosis which may have devastating and sometimes lethal consequences.

The clinical utility and importance of extended gene panel testing for clinically and genetically heterogeneous disorders such as epilepsy is undisputed. Knowledge of the precise genetic aetiology of a patient’s epilepsy can significantly alter therapeutic management and understanding the inheritance pattern of the genetic subtype informs genetic counselling for both the patient and the wider family [1]. However, the inclusion of inappropriate genes or omission of relevant genes can potentially lead to false-positive or missed diagnoses, respectively. The lack of consensus and international guidance for curation and inclusion of a gene in panels means that existing diagnostic panels often contain genes of research interest or those with historical (sometimes incomplete or inaccurate) evidence [2]. Although Genomics England PanelApp [3], ClinGen [2] and other similar initiatives are trying to address this issue, a review of current seizure panels offered by clinical laboratory studies revealed that this remains a significant concern. This is particularly important in epilepsy disorders where certain medications are either more effective in controlling seizures or contraindicated in patients with particular monogenic causes of disease [4]. Notable examples of this include the *SCN1A*-associated seizure disorders where commonly used sodium-channel-blocking medications carbamazepine, vigabatrin and lamotrigine should be avoided because they may worsen the condition by inducing and/or prolonging seizures [5]. Monoallelic variation of the alpha subunit of the sodium channel (*SCN1A*) gene is a well-established cause of a spectrum of seizure disorders that include simple febrile seizures, febrile seizures plus (FS+) and genetic epilepsy with febrile seizures plus (GEFS+). *SCN1A* variants also account for 70–80% of Dravet syndrome (DS), a debilitating autosomal dominant infantile-onset epileptic encephalopathy [6].

In 2009, Singh *et al*. described a large Utah family comprising 21 individuals affected by febrile/afebrile seizures clinically indistinguishable from the seizure phenotypes associated with *SCN1A* [7]. Their genetic studies identified a missense variant in the sodium channel protein type 9 subunit alpha (voltage-gated sodium channel Na_v_1.7; *SCN9A* NM_002977 c.1921A>T p.(Asn641Tyr)), thereafter referred to as *SCN9A* p.(Asn641Tyr), which cosegregated with the disease in all but one individual who did not have a history of seizures. Eleven variant carriers displayed a typical febrile convulsion history, with no seizures reported after age six years. In the remaining ten, afebrile seizures followed typical febrile convulsions, the majority of which resolved by age 16 years with only two progressing to intractable epilepsy. Inherited autosomal dominant forms of familial febrile seizures typically show reduced penetrance, with between 10 and 30% of individuals inheriting the familial gene variant remaining seizure free [8,9], whereas the penetrance of the seizure phenotype in this family was 95%. The authors then investigated a series of 92 unrelated patients with a personal history of febrile seizures with or without a family history of seizures, and identified a further five rare missense variants in *SCN9A*, with current allele frequencies ranging from 0 to 1.8% in the Genome Aggregation database (gnomAD v2.1.1, Table 1).

**Table 1:** Published heterozygous *SCN9A* variants proposed as a monogenic cause of seizure disorders shown alongside gnomAD allele frequency data. While some of these variants are rare or novel, a number [p.(Gln10Arg), p.(Ser490Asn), p.(Lys655Arg), p.(Ile739Val)] are present at notable allele frequency in heterozygous as well as homozygous state, inconsistent with them being causative of a monogenic seizure disorder. Additionally, the other reported variants were mostly defined in a single affected individual or small nuclear families in which limited, or no wider cosegregation studies could be performed. ^1^ gnomAD v2.1.1 non-neuro cohort.^.^Abbreviations: AC, Allele count; AF Allele frequency; AFS, Afebrile seizures; BECTS, benign partial epilepsy of childhood with centrotemporal spikes; FS, Febrile Seizures; GEFS+, generalised epilepsy with febrile seizures plus; GSW, generalised spike wave; Hom. Homozygous individuals; IGE, idiopathic generalised epilepsy; NGS, Next-generation sequencing; PCR, polymerase chain reaction. TLE, temporal lobe epilepsy.

GRCh38 reference (rs number)	Genotype (NM_002977)	Phenotype	gnomAD^1^ AC (Hom.) AF	*SCN9A* variant familial segregation	Reference
*SCN9A Variants proposed in Singh et al. 2009 [7]*					
**Chr2:166284506T>A** **(rs121908918)**	c.1921A>T p.(Asn641Tyr)	FS, AFS, TLE	3 (0) 0.001%	Utah family variant	Singh *et al*. 2009 [7]
Chr2:166311573T>C (rs121908920)	c.184A>G p.(Ile62Val)	FS	6 (0) 0.003%	*none stated*	Singh *et al*. *2009* [7]
**Chr2:166306531G>T** **(rs121908921)**	c.446C>A p.(Pro149Gln)	FS	0	*none stated*	Singh *et al*. 2009 [7]
**Chr2:166286469C>T** **(rs58022607)**	c.1469G>A p.(Ser490Asn)	complex FS	4023 (198) 1.8%	*none stated*	Singh *et al*. 2009 [7]
**Chr2:166281786T>C** **(rs121908919)**	c.1964A>G p.(Lys655Arg)	FS, GSW, IGE	428 (0) 0.2%	*none stated*	Singh *et al*. 2009 [7]
		GEFS+		Inherited from an unaffected parent.	Alves *et al*. 2019 [17]
**Chr2:166280452T>C** **(rs182650126)**	c.2215A>G p.(Ile739Val)	FS, IGE	472 (1) 0.2%	*none stated*	Singh *et al*. 2009 [7]
*SCN9A variants proposed in subsequent publications*					
**Chr2:166311728T>C** **(rs267607030)**	c.29A>G p.(Gln10Arg)	GEFS+	25 (1) 0.01%	Inherited from an affected parent and present in an affected sibling.	Cen *et al*. 2017 [14]
**Chr2:166307014A>G**	c.319T>C p.(Tyr107His)	FS, AFS	0	Inherited from a parent of unknown affectation	Banfi *et al*. 2020 [13]
**Chr2:166303195G>T** **(rs201743233)**	c.796C>A p.(Leu266Met)	GEFS+	2 (0) <0.001%	Inherited from an unaffected parent.	Mulley *et al*. 2013 [18]
**Chr2:166293358C>T** **(rs765818027)**	c.980G>A p.(Gly327Glu)	BECTS	11 (0) 0.005%	Inherited from an unaffected parent, identified in affected sibling.	Liu *et al*. 2019 [15]
		GEFS+		Inherited from an affected parent.	Yang *et al*. 2018 [12]
**Chr2:166198900del** **(rs1353037253)**	c.5702_5706del p.(I1901fs)	GEFS+	10 (0) 0.005%	Inherited from an affected parent	Yang *et al*. 2018 [12]
**Chr2:166198733T>C** **(rs761742207)**	c.5873A>G p.(Tyr1958Cys)	GEFS+	2 (0) <0.001%	Inherited from an affected parent and identified in a further affected individual and one individual of unknown affection.	Zhang *et al*. 2020 [16]

The studies undertaken in the Utah family included genome-wide linkage analysis, which identified a ~10cM region of Chr2q24 that cosegregated with the disease followed by targeted analysis of candidate genes. The Chr2q24 linked region encompassed ~65 genes including the known epilepsy gene cluster (*SCN1A*, *SCN2A* and *SCN3A*). Dideoxy sequencing of *SCN1A*, *SCN2A*, *SCN3A*, *SCN7A*, *KCNH7* and *SLC4A10* was performed, alongside Agilent Human Genome comparative genomic hybridization (CGH) Microarray 4x44K (Agilent, Santa Clara, CA) analysis of the distal 10 Mb of the Chr2q24 region (*SCN1A*, *SCN2A*, *SCN3A*, *SCN7A* and *SCN9A*) and multiplex amplicon quantification (MAQ) copy number variant (CNV) analysis of *SCN1A*, none of which revealed any candidate causative variants. However, as certain difficult to identify genomic variants (e.g. deep intronic, structural variants or CNVs) may evade detection using these techniques [10], it remains possible that an undiscovered pathogenic variant in one of the known epilepsy associated genes within the Utah family locus, in particular *SCN1A* with which there is a close phenotypical fit, may be responsible for the condition.

Intending to confirm the role of SCN9A in seizure susceptibility, the authors next generated an Scn9a-Asn641Tyr knock-in mouse model. This revealed a significant increase in susceptibility to electrically induced seizures in homozygous, but not heterozygous, animals [7,11]. Following this study, other rare/novel *SCN9A* variants (Table 1) were reported as putative monogenic causes of disease in individuals and small families with familial febrile seizures [12,13], FS+ and GEFS+ [14–18] and as a modifier of Dravet syndrome, in some cases in the presence of accompanying *SCN1A* pathogenic variants [7,18] (Table 1). These studies, stemming from those of Singh *et al*., have widely led to the inclusion of *SCN9A* on epilepsy gene testing panels.

**Fig 1:**
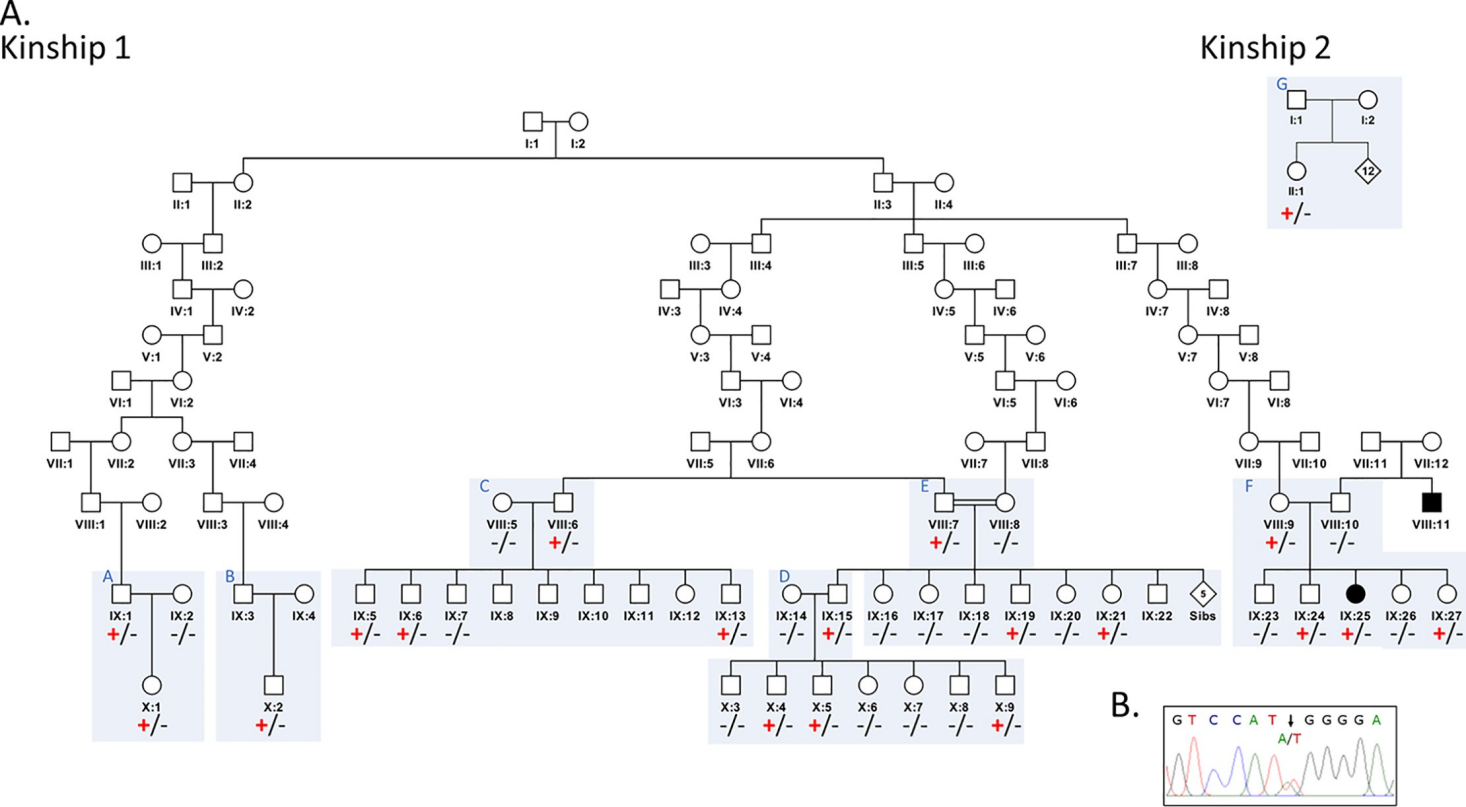
Family pedigrees showing *SCN9A* NM_002977 c.1921A>T p.(Asn641Tyr) genotype data. **(A).** Kinship 1: A simplified pedigree of the extended Amish family investigated, showing relationships between 18 distantly related individuals found to be heterozygous for the *SCN9A* p.(Asn641Tyr) variant. Individuals shaded black are affected with afebrile seizures. Kinship 2: An additional Amish family comprising of one individual with no history of seizures for whom exome data was available, found to be heterozygous for the *SCN9A* p.(Asn641Tyr) variant (II:1). Kinships 1 and 2 likely share common ancestry, but could not be connected through available records. Genotype is shown under individuals (variant: +, wild type: -). (B). Electropherogram showing the DNA sequence at the position of *SCN9A* c.1921A>T in a heterozygous individual.

Here we describe the serendipitous identification of the *SCN9A* p.(Asn641Tyr) variant within the Wisconsin Amish community, in which it is present at notable frequency in individuals with no personal or family history of febrile seizures. This Amish founder variant was identified as part of an ongoing study to characterise the genetic causes of inherited neurodevelopmental disorders present amongst the Wisconsin Amish and Mennonite communities (University of Arizona IRB (10-0050-01)), in which we investigated an Amish male infant presenting with dysmorphic facial features and global developmental delay (Fig 1. Kinship 1, X:9). Trio whole-exome sequencing identified a likely pathogenic *de novo* missense variant in *CHD4*, compatible with the child’s phenotype and the likely cause of the child’s syndromic presentation. In addition to the *CHD4* variant, our genomic studies also identified the *SCN9A* p.(Asn641Tyr) (Chr2(GRCh38):g.166284506T>A, NM_002977 c.1921A>T) variant inherited from the healthy father, who reported no history of febrile or afebrile seizures. Family extension studies were undertaken to investigate the relevance of the variant, in addition to cross-referencing these findings with our in-house Amish exome database alongside the comprehensive genealogical records of the Amish. These studies identified the *SCN9A* p.(Asn641Tyr) variant in a total of seven nuclear families (Fig 1) including three nuclear families (C,D and E) that closely interlink. In these families, further genetic studies confirmed the presence of the variant in 11 unaffected family members. Three other families (A,B and F) comprised of seven additional confirmed *SCN9A* p.(Asn641Tyr) variant carriers, all of whom interlink with the first three families through a 9th generation ancestral couple. A final nuclear family (G) could not be linked with available ancestral data. From these studies it is evident that the variant was transmitted through an additional minimum of 26 constitutive gene carrier parental couples and consequently will inevitably have been transmitted to hundreds of their offspring in whom genetic studies are not possible. The proband originally investigated in our study remains seizure free at two years and five months of age. Additionally, careful inspection of the available medical records across the wider extended Amish family and careful questioning of each individual carrier of the *SCN9A* p.(Asn641Tyr) variant and/or their parents, identified only one individual who carried the variant with a history of seizures. Importantly, this seven-year-old child (Kinship 1 IX:25) had a two-year history of left sided focal seizures not associated with loss of consciousness, normal development and no history of febrile convulsions. Metabolic testing and magnetic resonance imaging of the brain and spinal cord were unremarkable. While in a proportion of patients with focal seizures the disorder is associated with focal cortical dysplasia and/or monoallelic variants in the genes encoding the mTOR inhibitory GATOR1 complex [19], neither *SCN9A* nor *SCN1A* have been associated with this seizure type.

The high frequency of the *SCN9A* p.(Asn641Tyr) variant in the Amish involving hundreds of variant carriers with no history of seizure phenotypes is clearly inconsistent with it representing a highly penetrant cause of these conditions [7]. The benign nature of this variant is also consistent with our investigations in UK Biobank in which the variant was identified in two heterozygous carriers, neither of whom display a history of seizures. Further comparison of Amish *SCN9A* c.1921A>T p.(Asn641Tyr) carriers with those in UK Biobank shows that all eight Amish individuals for whom exome data was available (see methods in S1 Text and Fig 1 legend) and both UK Biobank *SCN9A* c.1921A>T p.(Asn641Tyr) carriers, share a rare synonymous variant in a closely linked gene; *TTC21B* (Chr2(GRCh38): g.165883959A>G NM_024753.4:c.3519T>C p.(Thr1173 =), rs115504901) situated ~400kb from *SCN9A*. The *TTC21B* variant has a European allele frequency of 0.4% (in gnomAD (v2.1.1) and a higher allele frequency of 5% in the Amish (in-house data). The complete co-occurrence of these two rare variants in the Amish and UK Biobank datasets in all individuals with *SCN9A* p.(Asn641Tyr) indicates that they likely occur *in cis*, and potentially all derive from an individual mutagenic event occurring in a single ancestral European founder in whom the *SCN9A* variant arose on the *TTC21B* haplotype.

The gnomAD database v.2.1.1 (non-neuro) currently identifies three non-Finnish European (NFE) heterozygous carriers of the *SCN9A* c.1921A>T p.(Asn641Tyr) variant at an overall allele frequency of 1.4x10^-5^ with a further five NFE carriers in gnomAD v3.0. GnomAD is an aggregated database of exome and genome data from unrelated individuals sequenced as part of various disease-specific and population genetic studies, it serves as a useful proxy population control dataset for severe early onset paediatric diseases and is utilised as an aide to genomic variant interpretation by research and diagnostic laboratories worldwide [20,21]. While it is not possible to draw meaningful conclusions about the pathogenicity of the *SCN9A* p.(Asn641Tyr) variant from gnomAD, this data confirms that it represents a low frequency variant present throughout the European population.

In the Amish, and many other community settings worldwide, particular genetic variants may become enriched and increase in allele frequency due to ancestral genetic bottleneck events, geographical isolation, community marriage patterns and large family sizes. This includes both pathogenic and benign variants for which increased allele frequency allows improved annotation and interpretation of pathogenicity [22,23]. The data presented here is a good example of this, repudiating the proposed autosomal dominant association of the p.(Asn641Tyr) *SCN9A* gene variant with seizure disorders.

The *SCN9A* variants identified as potentially pathogenic subsequent to p.(Asn641Tyr), include a number ([p.(Gln10Arg), p.(Ser490Asn), p.(Lys655Arg), p.(Ile739Val)]) which are present at population allele frequencies inconsistent with them being causative of a monogenic seizure disorder (Table 1 and S1 Table). Additionally, where this information is reported, these variants were all defined in either a single affected individual or small nuclear families, in which limited or no wider cosegregation studies could be performed and in which genomic studies were mostly relatively limited (Table 1 and S2 Table). Further evidence against an association between *SCN9A* and monogenic epilepsy is provided by a recent study of 31,058 parent-offspring trios, in which ~25% of probands had epilepsy/history of seizures. This study found no significant enrichment of *de novo* variants in *SCN9A*, with no history of epilepsy reported in the two *de novo SCN9A* variant carriers where this information was available [24]. Further to this, independent GWAS studies that show multiple significant associations between SNPs associated with *SCN1A/SCN2A* and epilepsy and/or febrile seizures, fail to do so for *SCN9A* [25]. Additionally, our own *SCN9A* rare (allele frequency <1%) variant burden analysis, of Caucasian epilepsy cases versus controls in UK Biobank exome data, defined no enrichment of plausibly causative rare *SCN9A* variants (S3 Table; p = 0.398), nor a disease association with any single variant (after correction for multiple testing).

SCN9A is unlike the epilepsy-related VGSC-α subunit molecules SCN1A, SCN2A, SCN3A and SCN8A [26–28] each of which is expressed primarily in brain, whereas SCN9A is expressed primarily in peripheral nerves (S1 Fig.). These primarily brain-expressed genes are also constrained for missense alterations and disease associated missense variants are primarily clustered over the functionally important ion transporter domains; neither of these scenarios is applicable to SCN9A (S2 Fig.) Thus, a role for SCN9A in sensory perception and pain is more congruous with our findings. Indeed, multiple studies document *SCN9A* gene variants associated with neuropathic pain syndromes including primary erythromelalgia, small fibre neuropathy and congenital insensitivity to pain [29–32], all of which is compatible with other predominantly peripheral expressed VGSC-α genes associated with similar phenotypes. However, the publications identifying an association between *SCN9A* and epilepsy have led to its widespread incorporation into monogenic inherited seizure disorder diagnostic testing panels [33], including Athena Diagnostics (MA, USA) [34], Blueprint Genetics (Finland) [35], Centogene (Germany) [36], Dynacare (Canada) [37], EGL Genetics (GA, USA) [38], Invitae (CA, USA) [39], Mayo Clinic Labs (MN & FL USA) [40]. The presence of *SCN9A* on these panels and its currently widely accepted status as an epilepsy disease gene, clearly presents a substantial risk of misdiagnosis to patients. This is of particular concern for genetic epilepsies in which a precise molecular diagnosis informs drug choice and a genetic misdiagnosis may have devastating and sometimes lethal consequences [13,41,42]. Thus given our findings, we consider ClinGen [33] and other expert groups reappraisal of the evidence regarding the role of *SCN9A* in monogenic seizure phenotypes to be of extreme importance and urgency, so as to refute this association and mitigate future harms.

## Ethics statement

The studies detailed in this manuscript were reviewed and approved by the University of Arizona IRB—1000000050. Written informed consent was obtained from participants or their parents.

## Supporting information

S1 Text Supplementary methods. (DOCX)

S1 Table UK Biobank allele frequencies for the *SCN9A* variants in Table 1. Allele frequencies are calculated from Biobank exome data (SPB pipeline), available for 49,959 individuals at the time of publication. Variant frequencies are separated by presence or absence of epilepsy as defined in our methods (above). All *SCN9A* variants examined are more frequent in controls than cases except p.(Ile739Val), where there is no significant difference between cases and controls (2 sided Fisher’s exact test p = 0.51)
(DOCX)

S2 Table Heterozygous *SCN9A* variants proposed as a monogenic cause of seizure disorders in subsequent publications, including the testing methodology employed. Abbreviations: AC, Allele count; aCGH, array comparative genomic hybridization AF Allele frequency; BECTS, benign partial epilepsy of childhood with centrotemporal spikes; FS, Febrile Seizures; GEFS+, generalised epilepsy with febrile seizures plus; Hom. Homozygous individuals; NGS, Next-generation sequencing; TLE, temporal lobe epilepsy. ^1^ gnomAD v2.1.1 non-neuro cohort.
(DOCX)

S3 Table Rare variant burden analysis in UK Biobank. Allele frequencies of *SCN9A* variants predicted to have an impact on SCN9A amino acid sequence were compared between cases and controls, with a small but not significant increase observed in controls (1 sided Fisher’s exact test p = 0.398).
(DOCX)

S1 Fig Sodium Voltage-Gated Channel Alpha Subunit gene family expression data. Expression data reproduced from the Genotype-Tissue Expression (GTEx) portal (www.gtexportal.org) for nine of the ten VGSC-α genes. Unlike the epilepsy-related VGSC-α genes (SCN1A, SCN2A, SCN3A and SCN8A), SCN9A is expressed primarily in peripheral nerves.
(DOCX)

S2 Fig Variant clustering in *SCN9A* alongside other VGSC-α genes (SCN1A and SCN3A) associated with epilepsy. *Top of genogram* (VGSC-α domains) shows the domain structure of VGSC-α genes, with four ion transporter Pfam domains (blue boxes) separated by interspersed disordered regions (beige boxes). *Beneath*, *SCN9A* is shown alongside the two VGSC-α genes that are both associated with epilepsy and have Pfam annotated ion channel domains (*SCN1A* and *SCN3A*), with the amino acid position of each domain indicated in each molecule (‘positions’ row). The number of ClinVar pathogenic annotated variants (with at least one pathogenic / likely pathogenic annotation) in each is shown below each domain. The four domains with the highest variant density are indicated by a darker shade. For *SCN1A* (which has a large number of disease-associated variants described) this is also calculated per amino acid to aid interpretation of the mutation load relative to the size of each of domain. Variants in *SCN9A* are separated into those associated with pain syndromes, and putative variants proposed to be associated with seizures; those associated with both are recorded twice, and those with no phenotypic description have been excluded. This shows that the epilepsy-related VGSC-α genes (*SCN1A*, *SCN3A*) display significant spatial clustering of putative disease-associated variants within regions known to be crucial for molecular function, in particular the ion transporter domain regions (shown by dark blue shading) and the interlinking regions between transporter domains 3 and 4 (shown by dark beige shading). For *SCN9A*, the expected clustering of variants in functionally important regions is only seen for pain-associated (erythromelalgia and paroxysmal pain) phenotypes with variants proposed to be associated with seizure disorder phenotypes displaying no spatial clustering, consistent with a benign nature.
(DOCX)

## References

[1] Kearney H, Byrne S, Cavalleri GL, Delanty N. Tackling Epilepsy With High-definition Precision Medicine: A Review. JAMA Neurol. 2019;76(9):1109–1116 10.1001/jamaneurol.2019.2384 31380988

[2] Strande NT, Riggs ER, Buchanan AH, Ceyhan-Birsoy O, DiStefano M, Dwight SS, et al Evaluating the Clinical Validity of Gene-Disease Associations: An Evidence-Based Framework Developed by the Clinical Genome Resource. Am J Hum Genet. 2017;100(6):895–906 10.1016/j.ajhg.2017.04.015 28552198 PMC5473734

[3] Martin AR, Williams E, Foulger RE, Leigh S, Daugherty LC, Niblock O, et al PanelApp crowdsources expert knowledge to establish consensus diagnostic gene panels. Nature genetics. 2019;51(11):1560–5 10.1038/s41588-019-0528-2 31676867

[4] Balestrini S, Sisodiya SM. Pharmacogenomics in epilepsy. Neurosci Lett. 2018;667:27–39 10.1016/j.neulet.2017.01.014 28082152 PMC5846849

[5] Wilmshurst JM, Gaillard WD, Vinayan KP, Tsuchida TN, Plouin P, Van Bogaert P, et al Summary of recommendations for the management of infantile seizures: Task Force Report for the ILAE Commission of Pediatrics. Epilepsia. 2015;56(8):1185–97 10.1111/epi.13057 26122601

[6] Wheless JW, Fulton SP, Mudigoudar BD. Dravet Syndrome: A Review of Current Management. Pediatric neurology. 2020;107:28–40 10.1016/j.pediatrneurol.2020.01.005 32165031

[7] Singh NA, Pappas C, Dahle EJ, Claes LR, Pruess TH, De Jonghe P, et al A role of SCN9A in human epilepsies, as a cause of febrile seizures and as a potential modifier of Dravet syndrome. PLoS genetics. 2009;5(9):e1000649 10.1371/journal.pgen.1000649 19763161 PMC2730533

[8] Mantegazza M, Gambardella A, Rusconi R, Schiavon E, Annesi F, Cassulini RR, et al Identification of an Nav1.1 sodium channel (SCN1A) loss-of-function mutation associated with familial simple febrile seizures. Proceedings of the National Academy of Sciences of the United States of America. 2005;102(50):18177–82 10.1073/pnas.0506818102 16326807 PMC1312393

[9] Bonanni P, Malcarne M, Moro F, Veggiotti P, Buti D, Ferrari AR, et al Generalized epilepsy with febrile seizures plus (GEFS+): clinical spectrum in seven Italian families unrelated to SCN1A, SCN1B, and GABRG2 gene mutations. Epilepsia. 2004;45(2):149–58 10.1111/j.0013-9580.2004.04303.x 14738422

[10] Møller RS, Schneider LM, Hansen CP, Bugge M, Ullmann R, Tommerup N, et al Balanced translocation in a patient with severe myoclonic epilepsy of infancy disrupts the sodium channel gene SCN1A. Epilepsia. 2008;49(6):1091–4 10.1111/j.1528-1167.2008.01550.x 18294202

[11] Oakley JC, Kalume F, Yu FH, Scheuer T, Catterall WA. Temperature- and age-dependent seizures in a mouse model of severe myoclonic epilepsy in infancy. Proceedings of the National Academy of Sciences. 2009;106(10):3994–9 10.1073/pnas.0813330106 PMC2656193 19234123

[12] Yang C, Hua Y, Zhang W, Xu J, Xu L, Gao F, et al Variable epilepsy phenotypes associated with heterozygous mutation in the SCN9A gene: report of two cases. Neurological Sciences. 2018;39(6):1113–5 10.1007/s10072-018-3300-y 29500686

[13] Banfi P, Coll M, Oliva A, Alcalde M, Striano P, Mauri M, et al Lamotrigine induced Brugada-pattern in a patient with genetic epilepsy associated with a novel variant in SCN9A. Gene. 2020;754:144847 10.1016/j.gene.2020.144847 32531456

[14] Cen Z, Lou Y, Guo Y, Wang J, Feng J. Q10R mutation in SCN9A gene is associated with generalized epilepsy with febrile seizures plus. Seizure. 2017;50:186–8 10.1016/j.seizure.2017.06.023 28704742

[15] Liu Z, Ye X, Qiao P, Luo W, Wu Y, He Y, et al G327E mutation in SCN9A gene causes idiopathic focal epilepsy with Rolandic spikes: a case report of twin sisters. Neurological Sciences. 2019;40(7):1457–60 10.1007/s10072-019-03752-3 30834459

[16] Zhang T, Chen M, Zhu A, Zhang X, Fang T. Novel mutation of SCN9A gene causing generalized epilepsy with febrile seizures plus in a Chinese family. Neurol Sci. 2020; 41(7): 1913–1917. 10.1007/s10072-020-04284-x PMC7359139 32062735

[17] Alves RM, Uva P, Veiga MF, Oppo M, Zschaber FCR, Porcu G, et al Novel ANKRD11 gene mutation in an individual with a mild phenotype of KBG syndrome associated to a GEFS+ phenotypic spectrum: a case report. BMC Med Genet. 2019;20(1):16 10.1186/s12881-019-0745-7 30642272 PMC6332862

[18] Mulley JC, Hodgson B, McMahon JM, Iona X, Bellows S, Mullen SA, et al Role of the sodium channel SCN9A in genetic epilepsy with febrile seizures plus and Dravet syndrome. Epilepsia. 2013;54(9):e122–e6 10.1111/epi.12323 23895530

[19] Iffland PH 2nd, Carson V, Bordey A, Crino PB. GATORopathies: The role of amino acid regulatory gene mutations in epilepsy and cortical malformations. Epilepsia. 2019;60(11):2163–73 10.1111/epi.16370 31625153 PMC7155771

[20] Karczewski KJ, Francioli LC, Tiao G, Cummings BB, Alföldi J, Wang Q, et al Variation across 141,456 human exomes and genomes reveals the spectrum of loss-of-function intolerance across human protein-coding genes. bioRxiv. 2019:531210

[21] Lek M, Karczewski KJ, Minikel EV, Samocha KE, Banks E, Fennell T, et al Analysis of protein-coding genetic variation in 60,706 humans. Nature. 2016;536(7616):285–91 10.1038/nature19057 27535533 PMC5018207

[22] Jung KS, Hong K-W, Jo HY, Choi J, Ban H-J, Cho SB, et al KRGDB: the large-scale variant database of 1722 Koreans based on whole genome sequencing. Database. 2020;2020 10.1093/database/baaa030 PMC7190023 32348452

[23] Abouelhoda M, Faquih T, El-Kalioby M, Alkuraya FS. Revisiting the morbid genome of Mendelian disorders. Genome biology. 2016;17(1):235 10.1186/s13059-016-1102-1 27884173 PMC5123336

[24] Kaplanis J., Samocha K.E., Wiel L. et al Evidence for 28 genetic disorders discovered by combining healthcare and research data. Nature (2020). 10.1038/s41586-020-2832-5 33057194 PMC7116826

[25] Buniello A, MacArthur JAL, Cerezo M, Harris LW, Hayhurst J, Malangone C, et al The NHGRI-EBI GWAS Catalog of published genome-wide association studies, targeted arrays and summary statistics 2019. Nucleic acids research. 2019;47(D1):D1005–D12 10.1093/nar/gky1120 30445434 PMC6323933

[26] Mulley JC, Scheffer IE, Petrou S, Dibbens LM, Berkovic SF, Harkin LA. SCN1A mutations and epilepsy. Human mutation. 2005;25(6):535–42 10.1002/humu.20178 15880351

[27] Berkovic SF, Heron SE, Giordano L, Marini C, Guerrini R, Kaplan RE, et al Benign familial neonatal-infantile seizures: characterization of a new sodium channelopathy. Annals of neurology. 2004;55(4):550–7 10.1002/ana.20029 15048894

[28] Vanoye CG, Gurnett CA, Holland KD, George AL Jr., Kearney JA. Novel SCN3A variants associated with focal epilepsy in children. Neurobiol Dis. 2014;62:313–22 10.1016/j.nbd.2013.10.015 24157691 PMC3877720

[29] Faber CG, Hoeijmakers JGJ, Ahn H-S, Cheng X, Han C, Choi J-S, et al Gain of function Naν1.7 mutations in idiopathic small fiber neuropathy. Annals of neurology. 2012;71(1):26–39 10.1002/ana.22485 21698661

[30] Yang Y, Wang Y, Li S, Xu Z, Li H, Ma L, et al Mutations in SCN9A, encoding a sodium channel alpha subunit, in patients with primary erythermalgia. Journal of medical genetics. 2004;41(3):171–4 10.1136/jmg.2003.012153 14985375 PMC1735695

[31] Michiels JJ, te Morsche RHM, Jansen JBMJ, Drenth JPH. Autosomal Dominant Erythermalgia Associated With a Novel Mutation in the Voltage-Gated Sodium Channel α Subunit Nav1.7. Archives of neurology. 2005;62(10):1587–90 10.1001/archneur.62.10.1587 16216943

[32] Cox JJ, Reimann F, Nicholas AK, Thornton G, Roberts E, Springell K, et al An SCN9A channelopathy causes congenital inability to experience pain. Nature. 2006;444(7121):894–8 10.1038/nature05413 17167479 PMC7212082

[33] ClinGen. SCN9A - epilepsy 2018 [cited 2020 11/08/2020]. Available from: https://search.clinicalgenome.org/kb/genes/HGNC:10597.

[34] Athena Diagnostics. Epilepsy Advanced Sequencing and CNV Evaluation 2020 [21/10/2020]. Available from: https://www.athenadiagnostics.com/view-full-catalog/e/epilepsy-advanced-sequencing-and-cnv-evaluation.

[35] Blueprint Genetics. Comprehensive Epilepsy Panel [21/10/2020]. Available from: https://blueprintgenetics.com/tests/panels/neurology/comprehensive-epilepsy-panel/.

[36] Centogene. Epilepsy Panel [21/10/2020]. Available from: https://www.centogene.com/science/centopedia/ngs-panel-genetic-testing-for-generalized-epilepsy-with-febrile-seizures.html

[37] Dynacare. Neurosure Epilepsy Gene Panel: Comprehensive (Ontario) [21/10/2020]. Available from: https://www.dynacare.ca/specialpages/secondarynav/find-a-test/nat/neurosure%C2%A0epilepsy%C2%A0gene%C2%A0panel-%C2%A0comprehensive.aspx?sr=ONT&st=.

[38] EGL Genetics. Epilepsy and Seizure Disorders Panel: Sequencing and CNV Analysis [21.10.2020]. Available from: https://www.egl-eurofins.com/tests/MEPI1.

[39] Invitae. Invitae Epilepsy Panel [21/10/2020]. Available from: https://www.invitae.com/en/physician/tests/03401/.

[40] Mayo Clinic Labs. Targeted Genes and Methodology Details for Epilepsy/Seizure Genetic Panels 2019 [21/10/2020]. Available from: https://www.mayocliniclabs.com/it-mmfiles/Targeted_Genes_and_Methodology_Details_for_Epilespy_Genetic_Panels.pdf.

[41] Helbig I, Ellis CA. Personalized medicine in genetic epilepsies–possibilities, challenges, and new frontiers. Neuropharmacology. 2020:107970 10.1016/j.neuropharm.2020.107970 32413583

[42] Williams v Quest Diagnostics, Inc.: United States District Court for the District Of South Carolina Columbia Division; 2018 p. 432.

