# Supplementary material for: No association between SCN9A and monogenic human epilepsy disorders
Source: PLoS Genet. 2020 Nov 20;16(11):e1009161. doi: 10.1371/journal.pgen.1009161 (PMC7717534; doi:10.1371/journal.pgen.1009161)
Supplement: S1 Text — (DOCX) [file pgen.1009161.s001.docx]

# **S1 Text: Supplemental paper**

# **No association between SCN9A and monogenic human epilepsy disorders**

James Fasham^1,2^, Joseph S Leslie^1^, Jamie W Harrison^1,3^, James Deline^4^, Katie B. Williams^5^, Ashley Kuhl^5^, Jessica Scott Schwoerer^5^, Harold E Cross^6^, Andrew H Crosby^1,*^, Emma L Baple^1,2,*^

**Affiliations**

1. RILD Wellcome Wolfson Centre, University of Exeter Medical School, Royal Devon & Exeter NHS Foundation Trust, Barrack Road, Exeter, UK
2. Peninsula Clinical Genetics Service, Royal Devon & Exeter Hospital, Gladstone Road, Exeter, UK
3. University of Exeter, Department of Biosciences, Exeter, UK
4. Center for Special Children, La Farge Medical Clinic-VMH, La Farge, Wisconsin, USA
5. Department of Pediatrics, University of Wisconsin, Madison, Wisconsin, USA
6. Department of Ophthalmology, University of Arizona College of Medicine, Tucson, Arizona, USA

**Supplemental methods**

**Genomic studies**

Blood/buccal samples were obtained with informed consent (University of Arizona IRB - 1000000050). DNA was extracted using standard techniques. Whole-exome sequencing (WES), was performed on nine individuals (VIII:9, VIII:10, IX:5, IX:7, IX:14, IX:15, IX:25, X1, X:9) using Agilent Sureselect Whole Exome v6 / Twist Human Core Exome targeting. Individual X:2 underwent clinical exome sequencing, using Illumina TruSight targeting.

In all cases read alignment (BWA-MEM (v0.7.17) was performed, mate-pairs fixed and duplicates removed (Picard v2.15.0), InDel realignment/base quality recalibration (GATK v3.7.0), single-nucleotide variant (SNV)/InDel detection (GATK HaplotypeCaller), annotation (Alamut Batch v1.8 or v1.10), and read depth (GATK DepthOfCoverage). Copy number variants were detected using both ExomeDepth

(<https://cran.r-project.org/web/packages/ExomeDepth/vignettes/ExomeDepth-vignette.pdf>

and <https://github.com/vplagnol/ExomeDepth>)

and SavvyCNV ([https://www.biorxiv.org/content/10.1101/617605v1](https://eur03.safelinks.protection.outlook.com/?url=https%3A%2F%2Fwww.biorxiv.org%2Fcontent%2F10.1101%2F617605v1&data=02%7C01%7CC.Salter%40exeter.ac.uk%7C7ecd4e5ca5e746ff9ad708d795aaca39%7C912a5d77fb984eeeaf321334d8f04a53%7C0%7C0%7C637142433085693844&sdata=d8RhBs83RYfbjNwlYa6QrtUeKl7%2ByLd5dRBSkdIuc9M%3D&reserved=0) and [https://github.com/rdemolgen/SavvySuite](https://eur03.safelinks.protection.outlook.com/?url=https%3A%2F%2Fgithub.com%2Frdemolgen%2FSavvySuite&data=02%7C01%7CC.Salter%40exeter.ac.uk%7C7ecd4e5ca5e746ff9ad708d795aaca39%7C912a5d77fb984eeeaf321334d8f04a53%7C0%7C0%7C637142433085703833&sdata=V3PuKGaNobdxYSFTuZ1uqjvTDVR9bP21fkmZVG6HsjY%3D&reserved=0)). This conforms to GATK Best Practices.

Dideoxy sequencing confirmation of the *SCN9A* NM_002977 c.1921A>T p.(Asn641Tyr) variant was undertaken using standard techniques. The *SCN9A* NM_002977 c.1921A>T p.(Asn641Tyr) variant was submitted to ClinVar ([www.ncbi.nlm.nih.gov/clinvar](http://www.ncbi.nlm.nih.gov/clinvar), accession SCV001371862).

**UK Biobank rare variant burden analysis**

The UK Biobank data for 49,953 participants with available exome sequence data called by the Regeneron Seal Point Balinese (SPB) pipeline, were used (1). This analysis was limited to those participants of Caucasian ancestry (41,249). The epilepsy phenotype was generated in Stata V16 (2) including as a case anyone with an ICD9/10 primary/secondary code containing any evidence of epilepsy (ICD9: 34509, 34510, 34519, 3452, 3453, 3454, 34550, 34559, 3457, 3459; ICD10: G40.0-9, G41.0, G41.1, G41.2, G41.8, G41.9) or any self-reported history of epilepsy.

The UK Biobank exome data were annotated with SnpEff 4.3t (3). All exonic and canonical splice site variants predicted to alter the SCN9A amino acid sequence, annotated in transcript NM 002977.3, with a frequency of <1% in both UK Biobank and GnomAD (4) were extracted and manually curated. Total raw variant numbers were compared in cases and control using a two-sided Fisher’s exact test, as previously described (5). In addition, variant frequencies were calculated, and exome wide association tests performed using Plink V1.9 (6).

**Supplemental References:**

1. Van Hout, C. V, Tachmazidou, I., Backman, J. D., Hoffman, J. X., Ye, B., Pandey, A. K., Gonzaga-Jauregui, C., Khalid, S., Liu, D., Banerjee, N., Li, A. H., Colm, O., Marcketta, A., Staples, J., Schurmann, C., Hawes, A., Maxwell, E., Barnard, L., … Lopez, A. (2019). Whole exome sequencing and characterization of coding variation in 49,960 individuals in the UK Biobank. BioRxiv, 572347. https://doi.org/10.1101/572347
2. StataCorp. 2019. *Stata Statistical Software: Release 16*. College Station, TX: StataCorp LLC.
3. "A program for annotating and predicting the effects of single nucleotide polymorphisms, SnpEff: SNPs in the genome of Drosophila melanogaster strain w1118; iso-2; iso-3.", Cingolani P, Platts A, Wang le L, Coon M, Nguyen T, Wang L, Land SJ, Lu X, Ruden DM. Fly (Austin). 2012 Apr-Jun;6(2):80-92. PMID: 22728672 [PubMed - in process]
4. Karczewski, K.J., Francioli, L.C., Tiao, G. et al. The mutational constraint spectrum quantified from variation in 141,456 humans. Nature 581, 434–443 (2020). https://doi.org/10.1038/s41586-020-2308-7
5. Cohen JC, Kiss RS, Pertsemlidis A, Marcel YL, McPherson R, Hobbs HH. Multiple rare alleles contribute to low plasma levels of HDL cholesterol. Science. 2004;305(5685):869-872. doi:10.1126/science.1099870
6. Purcell, S., Neale, B., Todd-Brown, K., Thomas, L., Ferreira, M. A. R., Bender, D., Maller, J., Sklar, P., de Bakker, P. I. W., Daly, M. J., & Sham, P. C. (2007). PLINK: A Tool Set for Whole-Genome Association and Population-Based Linkage Analyses. The American Journal of Human Genetics, 81(3), 559–575. https://doi.org/10.1086/51979
