# Supplementary material for: No association between SCN9A and monogenic human epilepsy disorders
Source: PLoS Genet. 2020 Nov 20;16(11):e1009161. doi: 10.1371/journal.pgen.1009161 (PMC7717534; doi:10.1371/journal.pgen.1009161)
Supplement: S1 Table — Allele frequencies are calculated from Biobank exome data (SPB pipeline), available for 49,959 individuals at the time of publication. Variant frequencies are separated by presence or absence of epilepsy as defined in our methods (above). All SCN9A variants examined are more frequent in controls than cases except p.(Ile739Val), where there is no significant difference between cases and controls (2 sided Fisher’s exact test p = 0.51) (DOCX) [file pgen.1009161.s002.docx]

**S2 Table: Heterozygous *SCN9A* variants proposed as a monogenic cause of seizure disorders in subsequent publications, including the testing methodology employed**

| Genotype (NM_002977) | Phenotype | gnomAD^1^  AC (Hom.)  AF | *SCN9A* variant familial segregation | Genetic testing strategy | Additional variants not excluded | Reference |
| --- | --- | --- | --- | --- | --- | --- |
| c.29A>G p.(Gln10Arg) | GEFS+ | 25 (1)  0.01% | Inherited from an affected parent and present in an affected sibling | **Proband only NGS panel***:* 480 epilepsy-related genes (including *SCN1A*) |  | Cen *et al*. 2017 [14] |
| c.319T>C  p.(Tyr107His) | FS, AFS | 0 | Inherited from an affected parent | **Proband only targeted NGS panel:** Cardiac and channelopathy-related genes, karyotype and aCGH | *de novo* **1.3 Mb duplication**, ***POLG*** and ***AKAP9*** variants | Banfi *et al*. 2020[13] |
| c.796C>A p.(Leu266Met) | GEFS+ | 2 (0)  <0.001% | Inherited from a parent of unknown affectation | **Dideoxy sequencing:**  *SCN1A/B*, *GABRG2*, *PCDH19* |  | Mulley *et al*. 2013 [18] |
| c.980G>A p.(Gly327Glu) | BECTS | 11 (0)  0.005% | Inherited from an unaffected parent and identified in an affected sibling | **Trio WES** |  | Liu *et al.* 2019 [15] |
|  | GEFS+ |  | Inherited from an affected parent | **Dideoxy sequencing:** *SCN1A* and common epilepsy genes |  | Yang *et al.* 2018 [12] |
| c.1964A>G p.(Lys655Arg) | GEFS+ | 428 (0)  0.2% | Inherited from an unaffected parent | **Trio WES and** **virtual gene panel analysis:**  21 epilepsy-related genes (including *SCN1A*) and aCGH | ***ANKRD11*** heterozygous nonsense | Alves *et al*. 2019 [17] |
| c.5702_5706del p.(I1901fs) | GEFS+ | 10 (0)  0.005% | Inherited from an affected parent | **Dideoxy sequencing:** *SCN1A* and common epilepsy genes |  | Yang *et al.* 2018 [12] |
| c.5873A>G  p.(Tyr1958Cys) | GEFS+ | 2 (0)  <0.001% | Inherited from an affected parent and identified in a further affected individual and one individual of unknown affection. | **Trio WES:**  *SCN1A* variants examined |  | Zhang *et al*. 2020 [16] |

Abbreviations: AC, Allele count; aCGH, array comparative genomic hybridization AF Allele frequency; BECTS, benign partial epilepsy of childhood with centrotemporal spikes; FS, Febrile Seizures; GEFS+, generalised epilepsy with febrile seizures plus; Hom. Homozygous individuals; NGS, Next-generation sequencing; TLE, temporal lobe epilepsy**.** ^1^ gnomAD v2.1.1 non-neuro cohort**.**
