# Supplementary material for: No association between SCN9A and monogenic human epilepsy disorders
Source: PLoS Genet. 2020 Nov 20;16(11):e1009161. doi: 10.1371/journal.pgen.1009161 (PMC7717534; doi:10.1371/journal.pgen.1009161)
Supplement: S2 Table — Abbreviations: AC, Allele count; aCGH, array comparative genomic hybridization AF Allele frequency; BECTS, benign partial epilepsy of childhood with centrotemporal spikes; FS, Febrile Seizures; GEFS+, generalised epilepsy with febrile seizures plus; Hom. Homozygous individuals; NGS, Next-generation sequencing; TLE, temporal lobe epilepsy. 1 gnomAD v2.1.1 non-neuro cohort. (DOCX) [file pgen.1009161.s003.docx]

**S2 Table: Heterozygous *SCN9A* variants proposed as a monogenic cause of seizure disorders in subsequent publications, including the testing methodology employed**

| Genotype (NM_002977) | Phenotype | gnomAD^1^  AC (Hom.)  AF | *SCN9A* variant familial segregation | Genetic testing strategy | Additional variants not excluded | Reference |
| --- | --- | --- | --- | --- | --- | --- |
| c.29A>G p.(Gln10Arg) | GEFS+ | 25 (1)  0.01% | Inherited from an affected parent and present in an affected sibling | **Proband only NGS panel***:* 480 epilepsy-related genes (including *SCN1A*) |  | Cen *et al*. 2017 [14] |
| c.319T>C  p.(Tyr107His) | FS | 0 | Inherited from an affected parent | **Proband only targeted NGS panel:** Cardiac and channelopathy-related genes, karyotype and aCGH | *de novo* **1.3 Mb duplication**, ***POLG*** and ***AKAP9*** variants | Banfi *et al*. 2020[13] |
| c.796C>A p.(Leu266Met) | GEFS+ | 2 (0)  <0.001% | Inherited from an unaffected parent | **Dideoxy sequencing:**  *SCN1A/B*, *GABRG2*, *PCDH19* |  | Mulley *et al*. 2013 [18] |
| c.980G>A p.(Gly327Glu) | BECTS | 11 (0)  0.005% | Inherited from an unaffected parent and identified in an affected sibling | **Trio WES** |  | Liu *et al.* 2019 [15] |
|  | GEFS+ |  | Inherited from an affected parent | **Dideoxy sequencing:** *SCN1A* and common epilepsy genes |  | Yang *et al.* 2018 [12] |
| c.1964A>G p.(Lys655Arg) | GEFS+ | 428 (0)  0.2% | Inherited from an unaffected parent | **Trio WES and** **virtual gene panel analysis:**  21 epilepsy-related genes (including *SCN1A*) and aCGH | ***ANKRD11*** heterozygous nonsense | Alves *et al*. 2019 [17] |
| c.5702_5706del p.(I1901fs) | GEFS+ | 10 (0)  0.005% | Inherited from an affected parent | **Dideoxy sequencing:** *SCN1A* and common epilepsy genes |  | Yang *et al.* 2018 [12] |
| c.5873A>G  p.(Tyr1958Cys) | GEFS+ | 2 (0)  <0.001% | Inherited from an affected parent and identified in a further affected individual and one individual of unknown affection. | **Trio WES:**  *SCN1A* variants examined |  | Zhang *et al*. 2020 [16] |

Abbreviations: AC, Allele count; aCGH, array comparative genomic hybridization AF Allele frequency; BECTS, benign partial epilepsy of childhood with centrotemporal spikes; FS, Febrile Seizures; GEFS+, generalised epilepsy with febrile seizures plus; Hom. Homozygous individuals; NGS, Next-generation sequencing; TLE, temporal lobe epilepsy**.** ^1^ gnomAD v2.1.1 non-neuro cohort**.**
