# Supplementary material for: No association between SCN9A and monogenic human epilepsy disorders
Source: PLoS Genet. 2020 Nov 20;16(11):e1009161. doi: 10.1371/journal.pgen.1009161 (PMC7717534; doi:10.1371/journal.pgen.1009161)
Supplement: S3 Table — Allele frequencies of SCN9A variants predicted to have an impact on SCN9A amino acid sequence were compared between cases and controls, with a small but not significant increase observed in controls (1 sided Fisher’s exact test p = 0.398). (DOCX) [file pgen.1009161.s004.docx]

**S3 Table: Rare variant burden analysis in UK Biobank**

|  | **Case Alleles** | **Control Alleles** | **Total Alleles** |
| --- | --- | --- | --- |
| **Variant alleles** | 30 | 2,835 | 2,865 |
| **Wild type alleles** | 465,996 | 37,149,705 | 37,615,701 |
| **Total**  **alleles** | 466,026 | 37,152,540 | 37,618,566 |

Allele frequencies of *SCN9A* variants predicted to have an impact on SCN9A amino acid sequence were compared between cases and controls, with a small but not significant increase observed in controls (two sided Fisher’s exact test p = 0.398).
