# Supplementary material for: No association between SCN9A and monogenic human epilepsy disorders
Source: PLoS Genet. 2020 Nov 20;16(11):e1009161. doi: 10.1371/journal.pgen.1009161 (PMC7717534; doi:10.1371/journal.pgen.1009161)
Supplement: S1 Fig — Expression data reproduced from the Genotype-Tissue Expression (GTEx) portal (www.gtexportal.org) for nine of the ten VGSC-α genes. Unlike the epilepsy-related VGSC-α genes (SCN1A, SCN2A, SCN3A and SCN8A), SCN9A is expressed primarily in peripheral nerves. (DOCX) [file pgen.1009161.s005.docx]

**S1 Fig.: Sodium Voltage-Gated Channel Alpha Subunit gene family expression data**


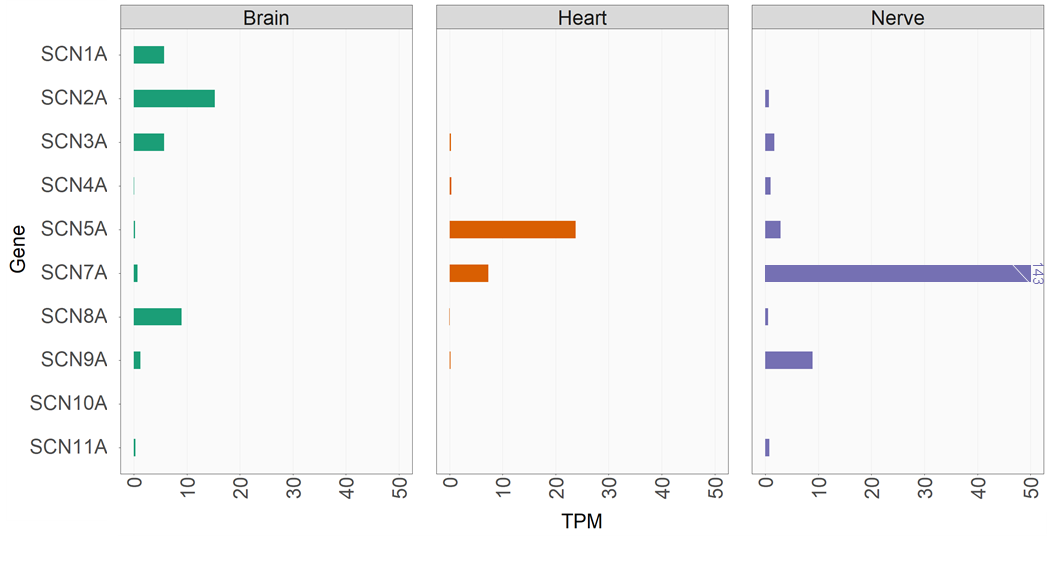


Expression data reproduced from the Genotype-Tissue Expression (GTEx) portal ([www.gtexportal.org](http://www.gtexportal.org)) for nine of the ten VGSC-α genes. Unlike the epilepsy-related VGSC-α genes (*SCN1A*, *SCN2A*, *SCN3A* and *SCN8A)*, *SCN9A* is expressed primarily in peripheral nerves.
