# Supplementary material for: No association between SCN9A and monogenic human epilepsy disorders
Source: PLoS Genet. 2020 Nov 20;16(11):e1009161. doi: 10.1371/journal.pgen.1009161 (PMC7717534; doi:10.1371/journal.pgen.1009161)
Supplement: S2 Fig — Top of genogram (VGSC-α domains) shows the domain structure of VGSC-α genes, with four ion transporter Pfam domains (blue boxes) separated by interspersed disordered regions (beige boxes). Beneath, SCN9A is shown alongside the two VGSC-α genes that are both associated with epilepsy and have Pfam annotated ion channel domains (SCN1A and SCN3A), with the amino acid position of each domain indicated in each molecule (‘positions’ row). The number of ClinVar pathogenic annotated variants (with at least one pathogenic / likely pathogenic annotation) in each is shown below each domain. The four domains with the highest variant density are indicated by a darker shade. For SCN1A (which has a large number of disease-associated variants described) this is also calculated per amino acid to aid interpretation of the mutation load relative to the size of each of domain. Variants in SCN9A are separated into those associated with pain syndromes, and putative variants proposed to be associated with seizures; those associated with both are recorded twice, and those with no phenotypic description have been excluded. This shows that the epilepsy-related VGSC-α genes (SCN1A, SCN3A) display significant spatial clustering of putative disease-associated variants within regions known to be crucial for molecular function, in particular the ion transporter domain regions (shown by dark blue shading) and the interlinking regions between transporter domains 3 and 4 (shown by dark beige shading). For SCN9A, the expected clustering of variants in functionally important regions is only seen for pain-associated (erythromelalgia and paroxysmal pain) phenotypes with variants proposed to be associated with seizure disorder phenotypes displaying no spatial clustering, consistent with a benign nature. (DOCX) [file pgen.1009161.s006.docx]

**S2 Fig.: Variant clustering in *SCN9A* alongside other VGSC-α genes (SCN1A and SCN3A) associated with epilepsy**


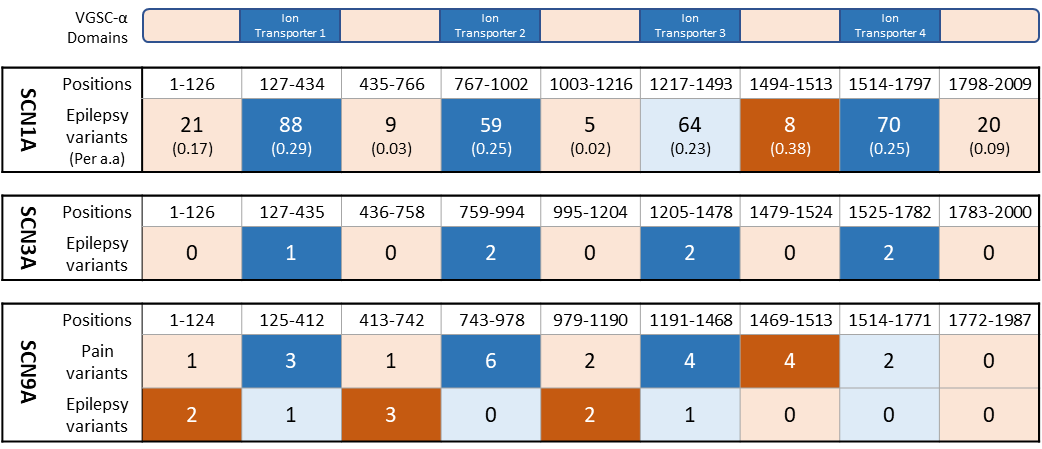


*Top of genogram* (VGSC-α domains) shows the domain structure of VGSC-α genes, with four ion transporter Pfam domains (blue boxes) separated by interspersed disordered regions (beige boxes). *Beneath*, *SCN9A* is shown alongside the two VGSC-α genes that are both associated with epilepsy and have Pfam annotated ion channel domains (*SCN1A* and *SCN3A*), with the amino acid position of each domain indicated in each molecule (‘positions’ row). The number of ClinVar pathogenic annotated variants (with at least one pathogenic / likely pathogenic annotation) in each is shown below each domain. The four domains with the highest variant density are indicated by a darker shade. For *SCN1A* (which has a large number of disease-associated variants described) this is also calculated per amino acid to aid interpretation of the mutation load relative to the size of each of domain. Variants in *SCN9A* are separated into those associated with pain syndromes, and putative variants proposed to be associated with seizures; those associated with both are recorded twice, and those with no phenotypic description have been excluded.

This shows that the epilepsy-related VGSC-α genes (*SCN1A*, *SCN3A*) display significant spatial clustering of putative disease-associated variants within regions known to be crucial for molecular function, in particular the ion transporter domain regions (shown by dark blue shading) and the interlinking regions between transporter domains 3 and 4 (shown by dark beige shading). For *SCN9A*, the expected clustering of variants in functionally important regions is only seen for pain-associated (erythromelalgia and paroxysmal pain) phenotypes with variants proposed to be associated with seizure disorder phenotypes displaying no spatial clustering, consistent with a benign nature.

.
